# ^18^F-FDG and ^11^C-4DST PET/CT for evaluating response to platinum-based doublet chemotherapy in advanced non-small cell lung cancer: a prospective study

**DOI:** 10.1186/s13550-019-0472-2

**Published:** 2019-01-16

**Authors:** Ryogo Minamimoto, Yuichiro Takeda, Masatoshi Hotta, Jun Toyohara, Kazuhiko Nakajima, Go Naka, Haruhito Sugiyama

**Affiliations:** 1 Division of Nuclear Medicine, Department of Radiology, National Center for Global Health and Medicine, 1-21-1, Toyama, Shinjyuku-ku, Tokyo, 162-8655 Japan; 2 Department of Respiratory Medicine, National Center for Global Health and Medicine, 1-21-1, Toyama, Shinjyuku-ku, Tokyo, 162-8655 Japan; 3 Research Team for Neuroimaging, Tokyo Metropolitan Institute of Gerontology, 1-1 Naka-cho, Itabashi-ku, Tokyo, 173-0022 Japan

**Keywords:** 4DST, FDG, NSCLC, Platinum doublet, PET/CT

## Abstract

**Background:**

4′-[Methyl-^11^C] thiothymidine (4DST) PET/CT provides DNA synthesis imaging, which represented a higher correlation with the proliferation in advanced non-small cell lung cancer (NSCLC) than that from imaging with FDG. The aim of this prospective study was to evaluate the potential of 4DST in early therapy monitoring for advanced NSCLC, and to compare the results with those from CT and FDG PET/CT.

**Results:**

Patients who had been pathologically diagnosed with advanced NSCLC and were scheduled to receive platinum-doublet chemotherapy (PT-DC) were eligible. PET/CT imaging with 4DST and with FDG, and CT were performed at baseline and after 2 cycles of PT-DC (interim). Patients were evaluated semi-quantitatively after the 2 cycles of PT-DC using several PET parameters, response evaluation criteria in solid tumors (RECIST) 1.1 based on CT measurements, European Organization for Research and Treatment of Cancer (EORTC) criteria and PET Response Criteria in Solid Tumors (PERCIST) 1.0 based on PET/CT measurements. Baseline measurement data and metabolic response were compared between patients with progression-free survival (PFS) > 4 months and ≤ 4 months, and PFS and overall survival (OS) were compared between patients with and without metabolic response measured with each of the different parameters, using Kaplan-Meier statistics and log-rank testing. A total of 22 patients were included in this study. For predicting PFS > 4 months and ≤ 4 months, metabolic tumor volume (MTV) of baseline 4DST showed the highest area under the curve (0.73), positive predictive value (80.0%), negative predictive value (66.7%), and accuracy (72.7%) among baseline measurement data and metabolic responses from 4DST PET/CT, FDG PET/CT, and CT. Kaplan-Meier curves and log-rank tests for PFS with MTV of baseline FDG and baseline 4DST, and for OS with MTV of baseline FDG and baseline TLG, and MTV of baseline 4DST revealed significant results.

**Conclusions:**

MTV of baseline 4DST PET/CT along with MTV of baseline FDG PET/CT represent promising predictors of PFS, and MTV of baseline 4DST PET/CT along with MTV and TLG of baseline FDG PET/CT are possible predictors of OS in patients with advanced NSCLC.

## Background

Platinum-based doublet chemotherapy (PT-DC) is the current standard of care as first-line therapy for patients with stage IIIB/IV non-small-cell lung cancer (NSCLC) [1]. In phase 3 randomized trials, PT-DC combinations have shown similar efficacy in terms of response rates, median progression-free survival (PFS), and overall survival (OS) [1–4]. Recently, second-line PT-DC has been reported to prolong survival in patients with advanced NSCLC [5]. Doublet chemotherapy is also recommended for patients who have not undergone testing for mutations or rearrangements. Furthermore, targeted therapies such as anti-angiogenic bevacizumab can be combined with PT-DC, except for patients with anaplastic lymphoma kinase (ALK) rearrangements and/or epidermal growth factor receptor (EGFR) mutations [1]. Immunogenic agents, particularly immune checkpoint inhibitors, have recently been combined with PT-DC for patients with rapid emergence of chemotherapy resistance [6–8].

Traditionally, treatment response has been assessed by response evaluation criteria in solid tumors (RECIST), which classifies effectiveness on the basis of tumor shrinkage from anatomical measurements, and positron emission tomography (PET) is used to identify new lesions in the revised guideline (RECIST version 1.1). However, tumor shrinkage tends to be followed by several metabolic changes related to tumor aggressiveness, with these changes induced by the treatment. PET allows for non-invasive monitoring of biology. In particular, 2-deoxy-2-[^18^F]fluoro-D-glucose (FDG) has been reported as a reliable tool for early prediction of response or resistance to treatment [9].

Tumor cell proliferation is a biological factor that is intimately related to malignancy, and has prognostic relevance in various malignancies, including NSCLC [10]. The thymidine analogue 3′-[^18^F]fluoro-3′-deoxythymidine (FLT) was reported to have great potential for indicating lung-tumor response or resistance to therapy [11–17]. FLT uptake reflects the activity of the enzyme thymidine kinase-1 (TK1) in the pyrimidine salvage pathway. A thymidine analog, carbon-11-labeled 4′-thiothymidine ([^11^C] 4DST (originally designated as [^11^C] S-dThd) is a cell proliferation imaging agent based on the mechanism of its incorporation into DNA [18–20]. In our previous report, 4DST PET/computed tomography (CT) demonstrated great potential for proliferation imaging in lung cancer and renal cell cancer that correlated highly with Ki-67 [21, 22]. Subsequently, we reported that 4DST PET/CT is sensitive to detecting mediastinal lymph node metastasis in NSCLC and may be helpful in predicting the prognosis of NSCLC [23].

We hypothesized that 4DST would offer a potent PET tracer for predicting the therapeutic effect of PT-DC. The objective of the current study was to observe the potential of 4DST PET/CT for predicting patient prognosis from baseline data, and interim response assessment, among NSCLC patients treated with PT-DC, compared to CT, FDG PET/CT, and several oncological response assessment criteria in current use.

## Methods

### Characteristics participants

This prospective study was approved by the local institutional review board of our hospital, and written informed consent was obtained from all patients prior to enrollment. Between October 2011 and June 2016, patients with advanced-stage NSCLC were recruited into the study. Inclusion criteria were (1) pathologically diagnosed NSCLC; (2) inability to perform treatment using radiation therapy with Union for International Cancer Control (UICC) 7th clinical stage IIIB or IV, or recurrence after surgery; (3) at least one measurable target lesion; (4) age > 20 years; (5) Eastern Cooperative Oncology Group performance (ECOG) status of 0 or 1; (6) no history of receiving PT-DC or planning to change PT-DC regimen due to insufficient treatment response confirmed in first- or second-line therapy; (7) no history of surgical operation within 4 weeks; (8) no history of radiation therapy within 2 weeks; and (9) white blood cell count ≥ 3000 mm^3^, hemoglobin ≥ 9.0 g/dl, platelets ≥ 100,000/mm^3^, total bilirubin ≤ 1.5 mg/dl, AST ≤ 100 IU/l, ALT ≤ 100 IU/l, serum creatinine ≤ 1.2 mg/dl, and SpO_2_ ≥ 90%. Exclusion criteria were (1) symptomatic brain metastasis; (2) double cancer; (3) symptomatic superior vena cava syndrome; (4) symptomatic pleural effusion, and/or ascites and/or cardiac effusion; (5) spinal compression by tumor; (6) uncontrolled hypertension or diabetes; (7) active interstitial pneumonia; (8) PT-DC allergy; (9) concurrent steroid therapy; or (10) pregnancy.

### Treatment

Two cycles of PT-DC were administered to all participants. Fifteen patients received PT-DC as first-line therapy, four patients as second-line therapy, and three patients as third-line therapy. Nine patients had received platinum plus tegafur/gimeracil/oteracil (TS-1), eight patients had received platinum plus pemetrexed, four patients had received platinum plus docetaxel, and one patient had received platinum plus gemcitabine. The median number of chemotherapy cycles was 4 (range, 2–5).

### Patient follow-up

The presence of a recurrent lesion was determined clinically based on the results of repeated contrast-enhanced (CE) chest and abdominal CT and CE brain magnetic resonance imaging (MRI) every 2–3 months for the first year, then every 4–6 months.

### Image acquisition

CE CT, FDG PET/CT, and 4DST PET/CT scans were performed within 20 days before initiation of PT-DC therapy (baseline) and after completing 2 cycles of PT-DC (interim). Multidetector-row CT (SOMATOM Definition: SIEMENS Health Care, or Discovery Revolution: GE Healthcare, or Aquilion One: Toshiba Medical Systems) covering the thorax, abdomen, and pelvis was performed with intravenous contrast, and images were reconstructed using a slice thickness of 2.5 mm. An in-house cyclotron and automated synthesis system (F200; Sumitomo Heavy Industries) was used in accordance with the authorized procedure to synthesize FDG. The time between tracer injection and acquisition of data was 60 min for FDG, and 293 ± 60 MBq of FDG was administrated on average. Mean blood glucose level was 104 ± 18 mg/dl (range, 69–152 mg/dl) for baseline FDG PET/CT and 114 ± 16 mg/dl (range, 90–151 mg/dl) for interim FDG PET/CT. Synthesis of 4DST was performed as described previously [18]. The time between tracer injection and acquisition of data was 40 min for 4DST, and 358 ± 40 MBq of 4DST was administered on average. PET images were obtained using Discovery PET/CT 600 (GE Healthcare) or Biograph 16 (SIEMENS Health Care) after the patient had fasted for at least 6 h. Cross-calibration was performed between the two PET scanners, and the same PET/CT scanner was used for baseline and interim PET/CT scans for each patient. Low-dose CT was performed first and was used for attenuation correction and image fusion. Emission images were acquired in three-dimensional mode for 2.5 min/bed position. PET data were reconstructed using a Gaussian filter with an ordered subset expectation maximization algorithm (3 iterations and 16 subsets for Discovery PET/CT 600; and 3 iterations and 8 subsets for Biograph 16).

### Image analysis

All CT studies were reviewed in consensus by at least two radiologists (≥ 10 years of experience), and RECIST uni-dimensional measurements were recorded prospectively by the same radiologists. All PET/CT studies were reviewed in consensus by at least two nuclear medicine physicians (≥ 5 years of experience), who were blinded to the clinical data and the results of other imaging studies. On baseline FDG PET/CT, we measured maximum standardized uptake value (SUV_max_) for the lesion showing the most intense FDG uptake (highest SUV_max_), sum of SUV_max_ for up to six lesions showing the highest SUV_max_ (sum of SUV_max_), total metabolic tumor volume (MTV), and total lesion glycolysis (TLG). We defined positive 4DST uptake as visually higher than that of the surrounding background and referred to the CT portion of the PET/CT to identify the targeted lesions. On baseline 4DST PET/CT, we measured SUV_max_ of the lesion showing the most intense 4DST uptake (highest SUV_max_), sum of SUV_max_ for up to six lesions showing the highest SUV_max_ (sum of SUV_max_), and total MTV. For the 4DST PET/CT, total lesion proliferation (TLP) was defined as SUV_mean_ multiplied by MTV. Assessed lesions were regarded as “targeted lesions” on interim PET/CT. On the interim PET/CT, we measured highest SUV_max_, sum of SUV_max_ for up to six lesions showing the most intense FDG or 4DST activity, the percentage decrease in SUV_max_ of targeted lesion between baseline and interim PET/CT (%Δ highest SUV_max_), the percentage decrease in sum of SUV_max_ for the target lesions (%Δ sum of SUV_max_), TLG, the percentage decrease in TLG between baseline and interim PET/CT (%ΔTLG), TLP and percentage decrease in TLP between baseline and interim PET/CT (%ΔTLP), MTV and the percentage decrease in MTV between baseline and interim PET/CT (%ΔMTV). SUV_max_, TLG, TLP, and MTV of lesions were measured with the PETedge tool MIM encore version 6.6 software (MIM Software, Cleveland, OH), using a gradient-based tumor segmentation method that detects the steepest drop-off in SUV values to create the contour boundary automatically [24].

### Response assessment based on CT and PET

Response to chemotherapy using CECT as interim analysis was assessed based on RECIST 1.1. For the FDG PET/CT, assessment of metabolic response was conducted in accordance with the European Organization for Research and Treatment of Cancer (EORTC) [25] and PET Response Criteria in Solid Tumors (PERCIST) 1.0 guidelines [26].

### Statistical analysis

Data are expressed as mean ± standard deviation. Differences in measurements of target lesions between baseline and interim scans with FDG PET/CT (SUV_max_, sum of SUV_max_, SUL **[**SUV normalized by lean body mass], MTV, TLG) and 4DST PET/CT parameters (SUV_max_, sum of SUV_max_, MTV, and TLP), differences in measurements of reference organs with FDG PET/CT (liver SUL and liver SUL standard deviation), and differences in measurements for sums of uni-dimensional measurements of target lesions were compared using the Wilcoxon signed-rank test in cases with patients receiving both baseline PET and interim PET. According to National Comprehensive Cancer Network (NCCN) guidelines, evaluation of tumor response for NSCLC is set after 4–6 cycles of doublet chemotherapy when deciding to proceed with subsequent therapy or continue maintenance therapy [1]. We thus evaluated differences in CT, FDG, and 4DST parameters between PFS > 4 months and ≤ 4 months, to estimate the status of disease progression after 4 cycles of PT-DC. Differences in FDG and 4DST PET parameters between PFS > 4 months and ≤ 4 months were compared using the Mann-Whitney *U* test. Receiver operating characteristic (ROC) analysis was performed to determine optimal cutoff values for uptake on baseline PET and uptake reduction from baseline to interim PET in predicting PFS > 4 months and ≤ 4 months. Positive predictive value (PPV), negative predictive value (NPV), and accuracy were calculated based on the optimal cutoff value determined by ROC analysis, and also according to RECIST 1.1, EORTC, and PERCIST 1.0. For RECIST 1.1, cases interpreted as showing stable disease (SD) or progressive disease (PD) were classified as non-responders to PT-DC, and studies interpreted as showing complete response (CR) or partial response (PR) were classified as responders to PT-DC. For EORTC criteria and PERCIST 1.0, studies interpreted as showing stable metabolic disease (SMD) or progressive metabolic disease (PMD) were classified as non-responders to PT-DC, and studies interpreted as showing complete metabolic response (CMR) or partial metabolic response (PMR) were classified as responders to PT-DC. PFS was calculated from the start of treatment until the date of disease progression or last follow-up. OS was calculated from the start of treatment until the date of death or last follow-up. Analyses of PFS and OS were performed using Kaplan-Meier estimates and log-rank tests for CT, FDG, and 4DST parameters. Times to progression and death served as endpoints. Analyses were performed using STATA version 14. Two-tailed values of *p* < 0.05 were considered significant.

## Results

### Patient characteristics

Twenty-two consecutive patients (mean age, 69.8 ± 8.8 years; 5 females, 17 males) were enrolled in this study. Patient characteristics are shown in Table 1. All patients underwent baseline CT, 4DST, and FDG PET/CT. These patients were included in the analysis to assess relationships between baseline PET and both PFS and OS. Four patients could not receive interim PET/CT because of clinical deterioration resulting from chemotherapy. Consequently, 18 patients with baseline CT, 4DST, and FDG PET/CT and interim CT, 4DST, and FDG PET/CT were included in the analysis to assess relationships between baseline PET and prognosis.

**Table 1:** Patient characteristics

Variable	Value
Age	
Mean ± SD	69.8 ± 8.8
Sex	
Male	17
Female	5
Histology	
Squamous cell carcinoma	13
Adenocarcinoma	9
Clinical stage	
IIIB	6
IV	16
Induction of PD-TC	
First-line	15
Second-line	4
Third-line	3

*PD-TC* platinum-based doublet chemotherapy

The mean interval between baseline FDG and baseline 4DST, between baseline CT and baseline 4DST, and between baseline FDG and baseline CT was 6 days, 15 days, and 12 days, respectively. The mean interval between initiating chemotherapy and any baseline scan was 5 days. The mean interval between interim FDG and interim 4DST, between interim CT and interim 4DST, and between interim FDG and interim CT was 2 days, 4 days, and 3 days, respectively. The mean interval between baseline and interim FDG, 4DST, and CT was 54 days, 51 days, and 62 days, respectively. Median follow-up for progression-free patients was 118 days (range, 36–556 days). Twelve patients showed PFS ≤ 4 months (range, 36–122 days), and 10 patients showed PFS > 4 months (range, 126–556 days). Median follow-up for surviving patients was 429 days (range, 78–1455 days).

### Differences in CT, FDG, and 4DST PET/CT parameters

**Fig. 1:**
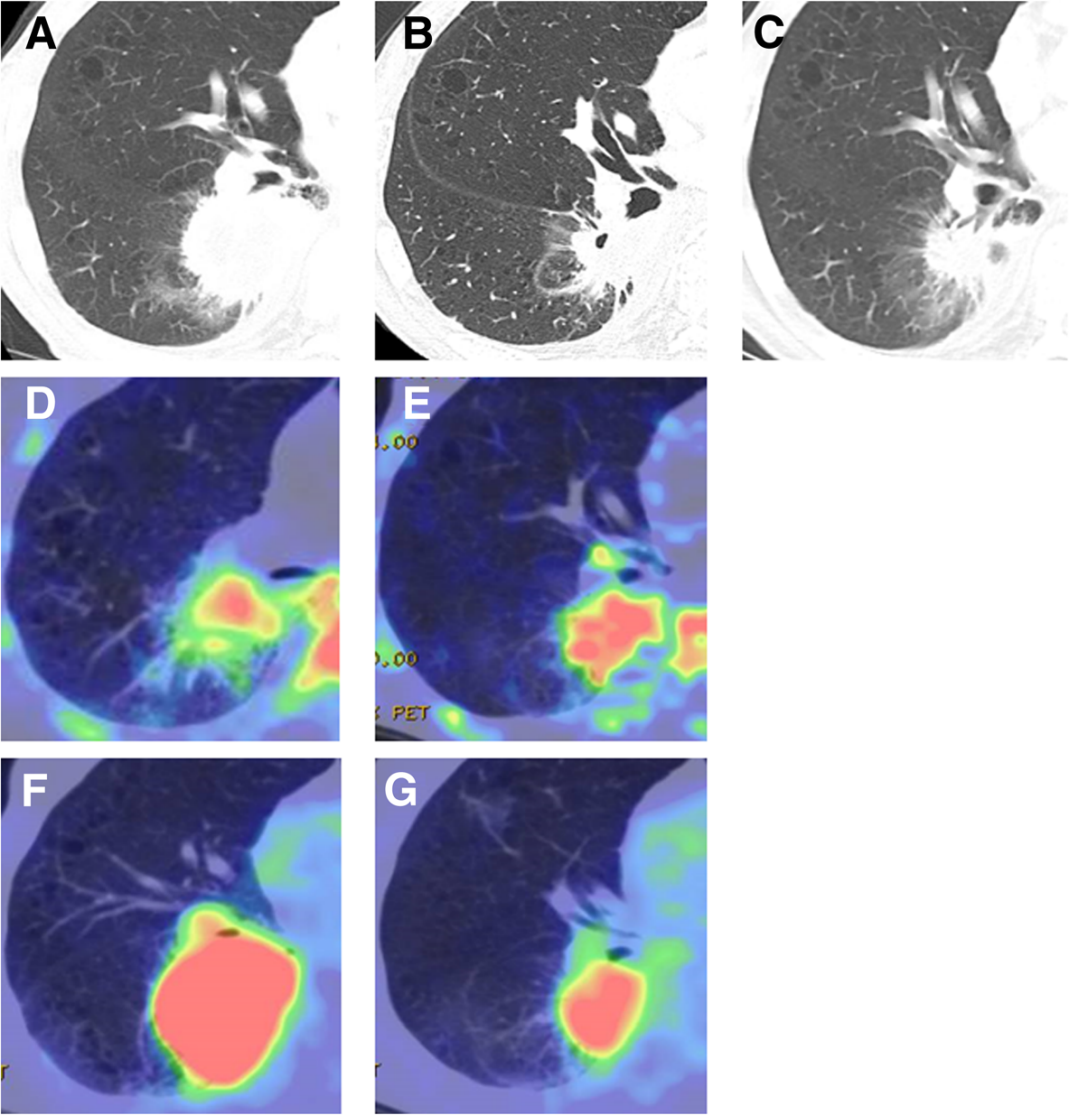
**a** Chest CT image at baseline. **b** Chest CT image after 2 cycles of platinum-based doublet chemotherapy (PT-DC). **c** Chest CT image after 5 cycles of PT-DC. **d** 4DST PET/CT image at baseline. **e** 4DST PET/CT image after 2 cycles of PT-DC. **f** FDG PET/CT image at baseline. **g** FDG PET/CT image after 2 cycles of PT-DC. Chest CT image at baseline shows lung lesion with a diameter of 55 mm in the right lower lobe (**a**), with intense uptake of both FDG (**f**) (SUV_max_ 15.8, MTV 81.0, TLG 672.5) and 4DST (**d**) (SUV_max_ 4.7, MTV 13.6, TLP 42.1). After 2 cycles of PT-DC, the diameter of lung cancer has decreased to 22 mm (**b**), categorized as partial response based on RECIST. However, 4DST uptake and uptake area were increased after 2 cycles of PT-DC (**e**) (SUV_max_ 4.9, MTV 18.2, TLP 56.4) compared to baseline 4DST PET/CT (**d**). FDG uptake and uptake area were decreased after 2 cycles of PT-DC (**g**) (SUV_max_ 7.0, MTV 25.0, TLG 86.4), categorized as partial metabolic response based on EORTC and PERCIST. After 5 cycles of PDC, the lung lesion showed regrowth with a diameter of 30 mm (**c**), indicating recurrence. Recurrence could be predicted earlier with 4DST PET/CT than with chest CT or FDG-PET/CT

Results are shown in Table 2. No significant differences were confirmed between baseline and interim assessments. Liver SUL and SD at baseline FDG and interim PET/CT displayed no significant difference. The results for differences in PET/CT parameters with PFS > 4 months and ≤ 4 months are shown in Table 3. No significant difference was observed in CT or PET parameters between PFS > 4 months and ≤ 4 months. Table 4 shows the result of ROC analyses and PPV, NPV, and accuracy of each PET parameter and several response assessment criteria. For predicting PFS > 4 months and ≤ 4 months, MTV of baseline 4DST showed the highest AUC (0.73), PPV (80.0%), NPV (67.7%), and accuracy (72.7%) among baseline measurement data and metabolic responses from 4DST PET/CT, FDG PET/CT, and CT. Represented images are shown in Fig. 1.

**Table 2:** Parameters of CT, FDG PET/CT, and 4DST PET/CT

Modality	Parameter	Baseline (*n* = 22)	Baseline (*n* = 18)	Interim CT or PET (*n* = 18)	*p* value
CT	Sum of uni-dimensional measurements of target lesion (cm)	71.9 ± 33.7	71.5 ± 28.7	61.5 ± 28.7	0.24
FDG	Highest SUV_max_	12.8 ± 5.0	13.7 ± 5.0	11.1 ± 7.0	0.36
	Sum of SUV_max_	35.2 ± 33.4	40.5 ± 34.8	29.0 ± 29.9	0.29
	MTV	101.6 ± 106.3	115.0 ± 112.5	77.7 ± 87.6	0.35
	TLG	618.1 ± 787.8	717.7 ± 838.4	408.1 ± 517.9	0.26
	SUL	8.9 ± 4.3	9.5 ± 4.4	7.5 ± 4.4	0.14
	Mean SUL of liver	1.8 ± 0.3	1.8 ± 0.3	2.0 ± 0.3	0.38
	SD of mean SUL of liver	0.2 ± 0.05	0.2 ± 0.05	0.2 ± 0.03	0.16
4DST	Highest SUV_max_	6.6 ± 2.6	6.1 ± 2.6	6.0 ± 2.7	0.73
	Sum of SUV_max_	19.6 ± 16.6	20.9 ± 17.8	19.7 ± 20.0	0.38
	MTV	145.9 ± 268.5	161.9 ± 286.3	80.6 ± 86.1	0.33
	TLP	507.6 ± 1107.3	553.9 ± 1187.1	232.6 ± 235.5	0.42

*MTV* metabolic tumor volume, *TLG* total lesion glycolysis, *SUL* lean body mass, *TLP* total lesion proliferation

**Table 3:** Difference in parameters with PFS > 4 months versus PFS ≤ 4 months

Modality	Parameters	PFS ≤ 4 months	PFS > 4 months	*p* value
CT	Sum of uni-dimensional measurements of target lesions (cm)	74.6 ± 42.8	69.2 ± 23.2	0.87
FDG	Highest SUV_max_	12.8 ± 5.6	11.7 ± 4.4	0.95
	Sum of SUV_max_	38.8 ± 41.6	28.6 ± 21.4	0.90
	MTV	138.0 ± 129.0	57.6 ± 43.5	0.19
	TLG	707.9 ± 701.7	292.3 ± 240.7	0.21
	SUL	10.0 ± 4.8	7.6 ± 3.3	0.28
	%Δhighest SUV_max_	− 12.2 ± 37.8	− 12.0 ± 80.7	0.42
	%Δsum of SUV_max_	− 19.2 ± 39.8	− 6.4 ± 85.1	0.79
	%ΔMTV	− 10.4 ± 59.4	− 29.1 ± 45.8	0.42
	%ΔTLG	− 10.9 ± 99.7	− 32.4 ± 79.0	0.33
	%ΔSUL	− 26.8 ± 47.5	− 34.4 ± 66.1	0.39
4DST	Highest SUV_max_	6.8 ± 2.4	5.7 ± 1.7	0.11
	Sum of SUV_max_	19.4 ± 17.8	22.8 ± 21.0	0.84
	MTV	127.7 ± 127.8	50.3 ± 29.2	0.06
	TLP	384.4 ± 371.0	158.4 ± 116.0	0.09
	%Δhighest SUV_max_	11.9 ± 37.3	− 5.6 ± 47.9	0.33
	%Δsum of SUV_max_	9.1 ± 49.5	− 18.6 ± 56.9	0.19
	%ΔMTV	− 18.9 ± 45.7	− 36.9 ± 52.4	0.33
	%ΔTLP	− 5.2 ± 77.9	− 32.9 ± 60.3	0.37

*MTV* metabolic tumor volume, *TLG* total lesion glycolysis, *SUL* standardized uptake value normalized by lean body mass, *TLP* total lesion proliferation

**Table 4:** Results of ROC analysis and PPV, NPV, and accuracy of predicting PFS > 4 months versus PFS ≤ 4 months

Modality	Parameter or interim assessments	AUC	PPV	NPV	Accuracy
CT	RECIST 1.1	0.56	60.0	66.7	61.1
FDG	Highest SUV_max_	0.51	60.0	57.1	59.1
	Sum of SUV_max_	0.52	41.7	30.0	36.4
	MTV	0.65	77.8	61.5	68.2
	TLG	0.62	66.7	53.8	59.1
	%Δhighest SUV_max_	0.61	66.7	55.6	61.1
	%Δsum of SUV_max_	0.53	61.5	60.0	61.1
	%ΔMTV	0.61	66.7	55.6	61.1
	%ΔTLG	0.64	66.7	66.7	66.7
	EORTC	0.60	70.0	55.6	66.7
	PERCIST	0.59	61.5	60.0	72.2
4DST	Highest SUV_max_	0.70	80.0	66.7	72.7
	Sum of SUV_max_	0.51	40.0	71.2	40.9
	MTV	0.73	80.0	66.7	72.7
	TLP	0.72	72.7	63.6	68.2
	%Δhighest SUV_max_	0.64	58.3	57.1	57.9
	%Δsum of SUV_max_	0.65	70.0	66.7	68.4
	%ΔMTV	0.59	60.0	55.6	57.9
	%ΔTLP	0.59	58.3	57.1	57.9

*MTV* metabolic tumor volume, *TLG* total lesion glycolysis, *SUL* standardized uptake value normalized by lean body mass, *TLP* total lesion proliferation

### Kaplan-Meier analysis for PFS and OS

**Fig. 2:**
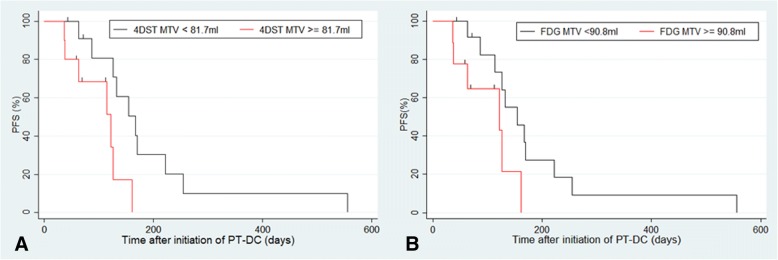
Kaplan-Meier analysis for progression-free survival (PFS) in patients with advanced non-small-cell lung cancer treated with platinum-based doublet chemotherapy (PT-DC). **a** PFS according to metabolic tumor volume (MTV) of baseline 4DST. **b** PFS according to MTV of baseline FDG

**Fig. 3:**
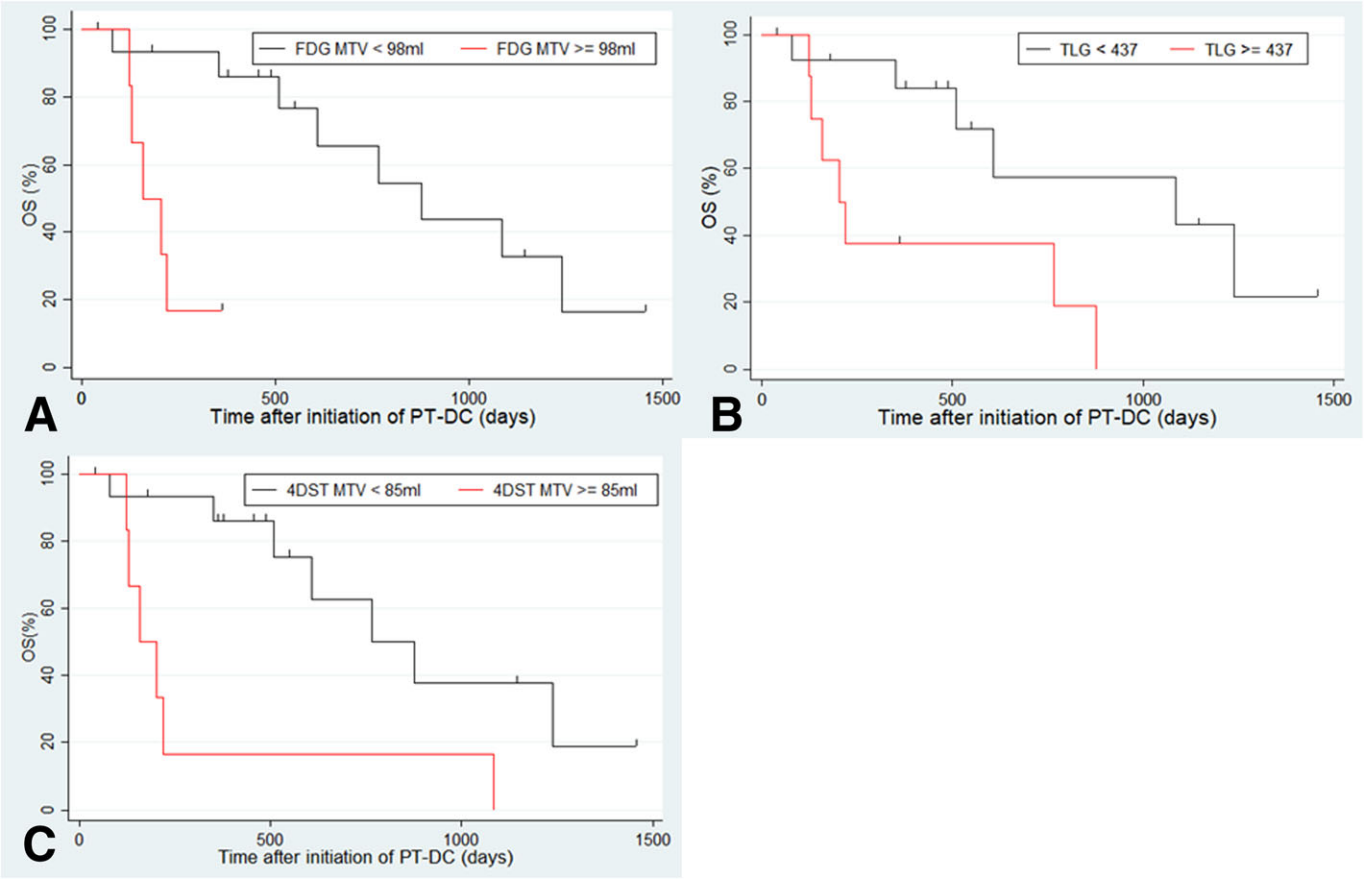
Kaplan-Meier analysis for overall survival (OS) in patients with advanced non-small-cell lung cancer treated with platinum-based doublet chemotherapy. (PT-DC). **a** OS according to metabolic tumor volume (MTV) of baseline FDG. **b** OS according to total lesion glycolysis (TLG) of baseline FDG. **c** PFS according to MTV of baseline 4DST

Kaplan-Meier curves for PFS with MTV of baseline FDG (*p* = 0.048) and baseline 4DST (*p* = 0.018) revealed significant results. No significant result was obtained from changes in parameters between baseline and interim PET for Kaplan-Meier curves of PFS (Table 5). The Kaplan-Meier curves for OS with MTV of baseline FDG (*p* = 0.02) and baseline TLG (*p* = 0.04), and MTV of baseline 4DST (*p* = 0.007) revealed significant results (Table 5, Figs. 2 and 3).

**Table 5:** Kaplan-Meier estimates and log-rank tests for PFS and OS in FDG and 4DST parameters

Modality	Parameter or interim assessments	*p* value	
		PFS	OS
CT	RECIST 1.1	0.79	0.28
FDG	Highest SUV_max_	0.63	0.13
	Sum of SUV_max_	0.84	0.99
	MTV	0.048	0.02
	TLG	0.12	0.04
	%Δ highest SUV_max_	0.07	0.94
	%Δsum of SUV_max_	0.46	0.69
	%ΔMTV	0.33	0.78
	%ΔTLG	0.26	0.62
	EORTC	0.53	0.85
	PERCIST	0.78	0.34
4DST	Highest SUV_max_	0.56	0.07
	Sum of SUV_max_	0.84	0.96
	MTV	0.018	0.007
	TLP	0.53	0.22
	%Δ highest SUV_max_	0.45	0.06
	%Δsum of SUV_max_	0.58	0.07
	%ΔMTV	0.26	0.29
	%ΔTLP	0.35	0.71

*MTV* metabolic tumor volume, *TLG* total lesion glycolysis, *TLP* total lesion proliferation

## Discussion

The present study evaluated 4DST PET/CT for predicting patient prognosis from baseline data and interim response assessment for NSCLC patient treated with PT-DC, compared to CT, FDG PET/CT, and several general oncological response assessment criteria (RECIST 1.1, EORTC, and PERCIST 1.0). Kaplan-Meier curves for PFS with MTV of FDG and 4DST and for OS with MTV of FDG and TLG, and MTV of 4DST revealed significant results.

Weber et al. studied 57 stage IIIB/IV NSCLC patients using FDG PET before and after the first cycle of a platinum-based chemotherapy. Metabolic response, defined as a reduction in tumor FDG uptake by > 20% after the first cycle of therapy, correlated significantly with overall responses as determined by RECIST [27]. Han et al. correlated OS with both baseline FDG uptake parameters as well as changes in these parameters after chemotherapy in advanced NSCLC. They concluded that a smaller baseline MTV and a greater decrease in MTV between baseline and interim PET images are significantly related to prolonged OS. This supports our premise that FDG PET could be used in both prognostic and predictive fashions in patients with NSCLC undergoing chemotherapy [28]. As in a previous study, our study results also showed MTV for FDG correlated with PFS and OS. FDG PET/CT is useful to assess response and changes in neoadjuvant chemotherapy for patients with resectable stage IB–IIIA lung cancers treated with PT-DC [29]. Our study covered stages III and IV did not have an impact on the change of PET parameters between baseline and interim PET. A potential limitation of FDG is false-positive uptake caused by metabolization at an inflammatory area or under a reactive state, so use of PET tracers specific to metabolic pathways in cancerous lesions have been expected to outperform FDG.

Early FDG PET and FLT PET have been used to predict PFS in 30 patients with advanced NSCLC treated with erlotinib, and the MTV of early FLT PET has been considered an effective predictor for monitoring response [15]. The reduction in FLT uptake after 7 days of therapy with gefitinib in 31 patients with NSCLC was reportedly highly predictive of both tumor response on CT at 6 weeks and PFS [16]. However, FLT uptake appeared to be influenced not only by proliferation, but also by changes in vascular permeability or perfusion [30]. A temporary increase in FLT uptake has been reported in lung cancer patients receiving chemoradiation, which may result in misleading therapeutic assessments [31]. FLT PET has been reported as failing to predict early therapeutic response in some types of cancer and/or therapy [32, 33]. Thus, whether FLT PET uptake actually captures growth inhibition (therapeutic response) may differ by type of cancer, chemotherapeutic drug, and treatment regimen. Moreover, while inhibition of proliferation is a key strategy, it is not always sufficient to achieve good outcomes due to the complexity of cancer.

Chandall et al. compared FDG and FLT in early response assessments of neoadjuvant therapy with PT-DC in patient with stages IB–IIIA resectable NSCLC, and they concluded that FLT PET imaging offers no significant advantage over FDG PET [34].

As a TK1-specific substrate, 4DST is resistant to catabolism by thymidine phosphorylase. Uptake of 4DST potentially reflects cell proliferation in a more precise manner than FLT uptake, and this uptake is unrelated to microvessel density [20]. According to one animal study, very low levels of 4DST uptake were observed in areas of subacute inflammation, which reflects the cell proliferation status of inflammatory tissues [35]. The dynamics of 4DST uptake differ between tumors and inflamed tissues. Uptake of 4DST in inflamed tissue was transiently increased during the subacute phase and subsequently decreased to normal levels. The uptake of 4DST in inflamed tissue was significantly increased on days 2–4 after turpentine injection, then decreased. By day 14, tracer uptake had returned to the day 1 level. The transient increase in 4DST uptake paralleled changes in Ki-67 labeling index in inflamed tissues [35]. These data indicate that the subacute phase needs to be avoided for proper evaluation of tumor response using 4DST PET. However, interim 4DST PET in our study was conducted just before starting the third cycle of PT-DC; in other words, we used the longest possible interval after the end of the second cycle, so tissue inflammation induced by chemotherapy was thought to have been minimized in this study.

Dynamic PET showed that the radioactivity in inflamed tissues peaked at 5 min after 4DST injection, then washed out over the next 15 min [36]. We started 4DST PET 40 min after injection of 4DST; therefore, 4DST uptake was thought to be less influenced by inflammatory changes that occurred in the tissue.

In our results, MTV of baseline 4DST offered the best parameter for predicting PFS > 4 month compared to ≤ 4 months. This fact may allow the use of 4DST as a good indicator of treatment strategy; that is, whether ongoing chemotherapy is to be changed or maintained. Theoretically, changes in PET parameters between baseline and interim measurements can reflect therapeutic effects on the target area that can be expected to be related to prognosis. However, our results showed no such parameters significantly related to prognosis. Our results identified MTV of baseline 4DST as the best parameter for predicting PFS and OS. Although 4DST showed some potential for predicting PFS and OS in patients with NSCLC treated using PT-DC, our result was considered to have little impact in terms of altering treatment plans.

As 4DST is a ^11^C-labeled PET tracer, use is limited to facilities with access to a cyclotron. One limitation for M staging with 4DST was anticipated to be physiological uptake by the liver, kidneys, and bone marrow. Since only a small number of patients were examined, a larger patient cohort needs to be examined to verify the diagnostic accuracy of 4DST PET for predicting PFS and OS. Although 4DST had a potential for predicting PFS and OS for patient of NSCLC treated with PT-DC, our result was considered to have little impact of altering the treatment plan. The standard of care has recently evolved toward giving chemotherapy alongside immunotherapy in the first-line setting. Further study should thus be conducted to evaluate whether 4DST and FDG have roles to play in recent trends such as PD-L1, PD-1, and combinations with existing treatment regimens for NSCLC. PET Edge is a gradient-based technique that detects the steepest drop-off in SUV values to automatically create contour boundaries. Wasik MW et al. showed that this method was statistically more accurate than a common thresholding technique [24]. However, validation with measurements of 4DST uptake has not been conducted.

## Conclusions

The present study evaluated 4DST PET/CT for predicting patient prognosis from baseline data and interim response in NSCLC patients treated using PT-DC, compared to CT, FDG PET/CT, and several general oncological response assessment criteria. Kaplan-Meier curves and log-rank tests for PFS with MTV of baseline FDG and baseline 4DST, for OS with MTV of baseline FDG and baseline TLG, and MTV of baseline 4DST revealed significant results. MTV of baseline 4DST PET/CT along with MTV of baseline FDG PET/CT offered promising predictors of PFS and MTV of baseline 4DST PET/CT, and MTV and TLG of baseline FDG PET/CT were possible predictors of OS in patients with advanced NSCLC.

## Abbreviations

4DST: 4′-[Methyl-^11^C] thiothymidine
ALK: Anaplastic lymphoma kinase
CE: Contrast enhanced
CR: Complete response
CT: Computed tomography
ECOG: Eastern Cooperative Oncology Group
EGFR: Epidermal growth factor receptor
EORTC: European Organization for Research and Treatment of Cancer criteria
FDG: 2-deoxy-2-[^18^F]fluoro-D-glucose
FLT: 3′-[^18^F]fluoro-3′-deoxythymidine
MRI: Magnetic resonance imaging
MTV: Metabolic tumor volume
NSCLC: Non-small cell lung cancer
OS: Overall survival
PD: Progressive disease
PERCIST: PET Response Criteria in Solid Tumors
PET: Positron emission tomography
PFS: Progression-free survival
PR: Partial response
PT-DC: Platinum-doublet chemotherapy
RECIST: Response evaluation criteria in solid tumors
ROC: Receiver operating characteristic
SD: Stable disease
SUV_max_: Maximum standardized uptake value
TK1: Thymidine kinase-1
TLG: Total lesion glycolysis
TLP: Total lesion proliferation
TS-1: Tegafur/gimeracil/oteracil
UICC: Union for International Cancer Control
